# Psychoneuroendocrinological effects of music therapy versus mindfulness in palliative care: results from the ‘Song of Life’ randomized controlled trial

**DOI:** 10.1007/s00520-021-06435-y

**Published:** 2021-08-06

**Authors:** Friederike Koehler, Jens Kessler, Martin Stoffel, Martin Weber, Hubert J. Bardenheuer, Beate Ditzen, Marco Warth

**Affiliations:** 1 Institute of Medical Psychology, Center for Psychosocial Medicine, University Hospital Heidelberg, Bergheimer Str. 20, 69115 Heidelberg, Germany; 2 Ruprecht-Karls-University Heidelberg, Heidelberg, Germany; 3 Center of Pain Therapy and Palliative Care Medicine, Department of Anesthesiology, University Hospital Heidelberg, Heidelberg, Germany; 4 Interdisciplinary Palliative Care Unit, III. Department of Medicine, University Medical Center of the Johannes Gutenberg University of Mainz, Mainz, Germany

**Keywords:** Music therapy, Cancer, Palliative care, Stress, Mindfulness, Oncology

## Abstract

**Purpose:**

Although research on psychosocial interventions in palliative care provided evidence for their effectiveness regarding patient-reported outcomes, few studies have examined their psychobiological effects yet. Therefore, the purpose of the present work as part of an overarching study was to investigate differential effects of music therapy versus mindfulness on subjective distress and both neuroendocrine and autonomic stress biomarkers.

**Methods:**

A total of 104 patients from two palliative care units were randomly assigned to three sessions of either music therapy or mindfulness. Before and after the second session (completed by 89 patients), participants rated their momentary distress and provided three saliva samples for cortisol and α-amylase analysis. Furthermore, photoplethysmography recordings were continuously assessed to calculate mean heart rate and heart rate variability. Data were analyzed using multilevel modeling of all available data and sensitivity analysis with multiply imputed data.

**Results:**

Between 67 and 75% of the maximally available data points were included in the primary analyses of psychobiological outcomes. Results showed a significant time*treatment effect on distress (*b* =  − 0.83, *p* = .02) indicating a greater reduction in the music therapy group. No interaction effects were found in psychobiological outcomes (all *p* > .05), but multilevel models revealed a significant reduction in cortisol (*b* =  − 0.06, *p* = .01) and mean heart rate (*b* =  − 7.89, *p* = .05) over time following either intervention.

**Conclusion:**

Findings suggest a beneficial effect music therapy on distress while no differential psychobiological treatment effects were found. Future studies should continue to investigate optimal stress biomarkers for psychosocial palliative care research.

****Trial Registration**:**

German Clinical Trials Register (DRKS)—DRKS00015308 (date of registration: September 7, 2018)

## Background

Palliative and supportive care aims at the relief of suffering in patients facing a life-threatening disease addressing their needs holistically on a physical, psychological, social, and spiritual level. Therefore, psychosocial interventions from various disciplines have been developed with a therapeutic focus on emotional, spiritual, or interpersonal consequences of a terminal disease and its symptoms, or more broadly, on the relief of stress.

The bio-psycho-social model assumes a reciprocal influence among all three levels contributing to health and disease. For instance, chronic psychosocial stress can affect cancer risk and tumor progression by facilitating inflammatory processes and weakening the immune system [1]. Additionally, the diagnosis and treatment of the illness itself may be highly strenuous, which can further increase stress-related symptoms [2]. In this context, psychosocial interventions were hypothesized to reduce stress [3] and to impact clinical outcomes via pathways on a biological, psychological, and social level [4, 5].

To monitor possible alterations in stress regulatory systems, cancer research has attended to cortisol and α-amylase as well as heart rate variability (HRV) as non-invasive markers of stress [6, 7]. In the presence of a stressor, the body responds by activating two neuroendocrine pathways involving the hypothalamus-pituitary-adrenal (HPA) and the sympathetic-adreno-medullary (SAM) system [1]. As the primary endpoint of the HPA axis, cortisol is released via the adrenal cortex, impacting fundamental physiological processes, such as metabolism and the immune and cardiovascular systems [2]. The SAM axis regulates the sympathetic responses to stress by initiating the release of norepinephrine and epinephrine via the adrenal medulla. At the salivary glands, these catecholamines were found to increase the secretion of the enzyme α-amylase into saliva [8]. While not always consistent, the majority of studies observed elevated cortisol and α-amylase levels in advanced cancer patients as well as flattened diurnal cortisol patterns compared to other diseases or healthy controls [6, 9–13].

To investigate the role of the parasympathetic nervous system (PNS) via the vagus nerve, HRV has been introduced as a cardiac index of autonomic flexibility based on the observation that variability in successive heartbeats mirrors the organism’s ability to flexibly adapt to environmental challenges [14]. High HRV has been associated with resilience, social engagement, well-being, and psychological flexibility [15] and can be increased by internal and external stimuli, such as mindfulness [16] or music [17]. With regard to oncological diseases, research found lower HRV compared to healthy participants [18] and provides evidence for HRV as a predictor of survival in advanced cancer patients [19].

Research investigating interventions based on cognitive behavioral therapy, music therapy, relaxation, mindfulness, and yoga demonstrated beneficial effects on neuroendocrine, autonomic, and immune parameters in cancer patients [20–23]. In palliative care settings or in patients nearing the end of life, however, there is a particular lack of research on the psychobiological effects of psychosocial interventions, possibly due to the patients’ weak health status, high medication intake, severe symptoms of xerostomia or nausea, and high attrition rates [24]. The available studies either focused on music therapy or mindfulness using brief and flexible intervention protocols considering the unique conditions in palliative care [25, 26]. Two studies found a decrease of perceived distress and heart rate, but no changes in HRV in response to brief and standardized mindfulness interventions [27, 28]. Another study on the effects of a music therapy showed a stronger increase in HRV, peripheral blood flow, and self-rated relaxation compared to prerecorded mindfulness [29, 30]. In a pilot study, palliative care patients receiving a single music therapy session reported increased existential well-being but showed no differences in cortisol levels compared to the control group [31]. In contrast, another pilot study with hospice patients observed a reduction in cortisol levels after a music intervention but lacked a control group [32].

The original aim of the present randomized controlled trial was to evaluate the efficacy of a three-session biographical music therapy intervention (‘Song of Life’; SOL) compared to a control relaxation/mindfulness treatment in palliative care [33]. The results reported in a previous publication showed significant beneficial pre-to-post intervention effects of music therapy on self-rated psycho-spiritual quality of life. [34]. Due to the paucity of research linking psychological with biological effects of psychosocial interventions in palliative care, we additionally aimed to explore stress biomarker trajectories in response to both interventions during the second session, in which a biographically meaningful song was played live to the patient. The present work will therefore focus on the psychobiological assessment of intervention effects during the second session (S2).

## Methods

### Study design

In a multicenter randomized controlled design, the present study had two trial conditions in parallel assignment: SOL music therapy plus usual care in the experimental group (EG) versus a relaxation/mindfulness intervention (RELAX) plus usual care as the control group (CG). The study was conducted at the University Palliative Care Unit at St. Vincentius Hospital, Heidelberg University Hospital, Germany, and the Interdisciplinary Palliative Care Unit at the University Medical Center of the Johannes Gutenberg University of Mainz, Germany. The trial was approved by the two responsible ethical review boards and was preregistered at the German Clinical Trials Registry (DRKS00015308). A study protocol with methods and procedures had been published prior to trial conduction [33].

### Participants

Eligible patients were 18 years or older, received specialized palliative treatment according to OPS 8–982/OPS 8-98e (German modification of International Classification of Procedures in Medicine; ICPM), or had an estimated life expectancy of < 12 months and could provide informed consent. Exclusion criteria were no proficiency in German language, a clinical estimation of life expectancy < 1 week, cognitive and auditory impairments, and psychiatric symptoms.

### Randomization, masking, and blinding

A computer-based block randomization sequence (block size = 8) was used to allocate participants with stratification by the study site before the commencement of the study and was unknown to the research staff conducting the study. Allocation concealment was achieved through opening sequentially numbered opaque envelopes after the participant had provided written informed consent and finished baseline assessment. For blinding of participants, we used an active control group while patients were not informed about the intervention under investigation. Blinding of therapists and outcome assessors was not feasible (single-blind).

### Procedures

Patients were asked to sign the consent form and to complete a baseline assessment. Research staff afterwards opened an envelope with the group assignment. Both interventions consisted of three 20–30-min sessions, each with a pre-to-post assessment of momentary distress.

**Fig. 1:**
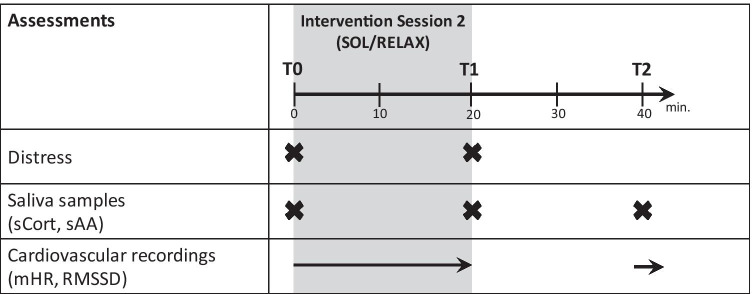
Timing of outcome assessments in intervention session 2. SOL “Song of Life” music therapy, RELAX relaxation intervention, T time point, sCort salivary cortisol, sAA salivary α-amylase, mHR mean heart rate, RMSSD root mean square of successive differences

The second session (S2) was complemented by psychobiological assessments (i.e., neuroendocrine and autonomic stress markers) as this session contained the live music performance. During this session, patients were asked to deliver 3 salivary samples in 20-min intervals for cortisol and α-amylase measurement, immediately before (T0) and after the session (T1) and at follow-up (T2, Fig. 1)), in order to capture biological stress gradients over time. A photoplethysmography (PPG) sensor (biosignalplux, Lisbon, Portugal) was placed on the index finger of the patient’s non-dominant hand to assess the cardiovascular response in a continuous recording during S2, as well as 20 min later as a follow-up 5-min segment (parallel to saliva sample T2). To address the impact of confounding factors, patients were asked to refrain from eating and drinking an hour before the session, if possible. Further, we assessed potentially confounding variables (e.g., eating, drinking, wake-up) in a short interview before and after each session and documented medication intake. After the last session (S3), research staff completed post-intervention outcome assessment.

### Interventions

Both interventions were carried out by two music therapists who were employed at the participating palliative care wards who were trained in all intervention procedures. The SOL music therapy in the EG began with a first session (S1) of conversation between therapist and patient aiming to determine a biographically meaningful and emotionally arousing song. In S2, the therapist sang this song live accompanied by guitar or e-piano and modified to a lullaby style (slow 3/4 or 6/8 rhythm), while the performance was audio-recorded. The therapist gave the edited recording to the patient in S3 and discussed feelings and memories by pre-defined questions. The translation of the song into triple or 6/8 rhythm was inspired by other music therapy techniques which have, for instance, been described for use in neonatal care [35]. The lullaby style in the songs aimed to support relaxation and a sense of security and containment. The SOL intervention needs to be carried out by a music therapist who received training in both the specific musical competency (e.g., translation of the song, adjusting the music to the patient’s breath) as well as therapeutic competency (e.g., building a trustworthy atmosphere, handling intense emotions).

Patients in the relaxation/mindfulness intervention (CG) participated in three standardized sessions of muscle relaxation (S1), mindful focus on the breath (S2), and imagery (S3), with a short inquiry at the end of each session. Techniques did not involve any musical, spiritual, or biographical themes. The target session S2 consisted of a standardized mindful breathing exercise, in which patients were instructed to become aware of different facets of the breath (e.g., duration, bodily sensations, breaks) non-judgementally. The 15-min exercise was followed by a debriefing asking for arising feelings and thoughts. Detailed intervention manuals have been published [34].

### Outcome measures

Before and after S2, patients were asked to rate momentary distress using a modified version of the NCCN Distress Thermometer [36] ranging from 0 (‘no distress’) to 10 (‘extreme distress’) [37].

Salivary cortisol (sCort) and α-amylase (sAA) were repeatedly assessed by means of saliva samples (T0–T2; Fig. 1). To minimize variance due to diurnal cortisol patterns, the second session always took place between 2 and 6 pm. Salivette® (Sarstedt, Nümbrecht, Germany) devices were used for saliva sampling. Patients were asked to chew on the synthetic swab for 1 min. Salivettes were later centrifuged, and the aliquoted saliva was stored in polypropylene vials at the laboratory of the Institute of Medical Psychology, Heidelberg, Germany. A commercially available enzyme-linked immunosorbent assay (ELISA; DES6611; Demeditec Diagnostics, Kiel, Germany) was used to analyze concentration of sCort (ng/ml). Concentrations of sAA (U/ml) were quantified using a kinetic colorimetric kit with reagents from Roche (Roche Diagnostics, Mannheim, Germany). The intra-assay coefficient of variation (CV) was 3.05% for sCort and 3.87% for sAA. The inter-assay CV was 3.82% for sCort and 8.37% for sAA.

In addition, continuous PPG recordings served to assess participants’ cardiac autonomic response in terms of beat-to-beat variations in heart rate. We derived inter-beat-intervals (IBI) between successive heartbeats in milliseconds for three time segments of 5-min duration corresponding to T0–T2 [38] (Fig. 1). Research commonly associates the root mean square of successive differences (RMSSD) with parasympathetic activity and ability to recover [39]. We thus used RMSSD as a marker of vagally mediated HRV and the mean heart rate (mHR) as a general biomarker of autonomic activity.

### Statistical analysis

Due to the hierarchical data structure (repeated observations on Level 1 nested in patients on Level 2), we performed multilevel modeling (MLM) in the statistical environment R. In an intention-to-treat approach, primary analysis was performed based on all available data (AAD). MLM parameters were obtained via maximum likelihood (ML) estimation with the R package ‘lme4’ [40], while *p* values for fixed effects were calculated via ‘lmerTest’. Separate multilevel models were computed to predict distress, sCort, sAA, mHR, and RMSSD. Based on visual inspections of variable distributions, sCort, sAA, and RMSSD were log transformed to approximate normality in the distribution of model residuals. Outliers beyond three standard deviations from the mean were excluded.

All outcome models included fixed predictors for time (0, 1, 2), treatment (contrast coded; 0 = RELAX, 1 = SOL), time*treatment, and study site (0, 1). If visual inspection of data revealed evidence for a non-linear trajectory over time, we added a quadratic polynomial for the ‘time’ variable (0, 1, 4), which was the case in mHR and RMSSD. If likelihood ratio tests revealed a significantly improved model fit with the quadratic term, this model was selected as final.

To test the hypotheses, we built random-intercept models with preselected covariates which were recommended in previous literature for psychobiological outcomes [24, 41–43]. These covariates included sex (0 = male, 1 = female) and age (years) for all outcomes as well as corticosteroid medication, sedative medication (0 = no intake, 1 = intake), and time since last meal (minutes) for sCort. All models were further estimated including a random slope of time to test for intraindividual variation. However, likelihood ratio tests comparing the nested models indicated no significantly improved model fit with an additional random effect of time, so random-intercept models were maintained. Both continuous predictors (age, time since last meal) were measured on level 2 and were centered on the grand mean. Each final model was graphically assessed for violations of central model assumptions (e.g., using qq-plots, or plots of residuals against predictors and fitted values).

Finally, we replicated the described models with multiply imputed data (MID) for sensitivity analyses with regard to missing data. To that end, we created sets of 20 multilevel imputations for each model and pooled the results with the R package ‘mitml’ [44]. Although analyses were explorative, based on literature and previous research [28, 29, 34], we postulated beneficial treatment effects of both interventions (decrease of distress, sCort, sAA, mHR, and increase of RMSSD) and hypothesized these effects to be significantly more pronounced in the music therapy group.

Sample size calculations were presented in the study protocol [33].

## Results

**Fig. 2:**
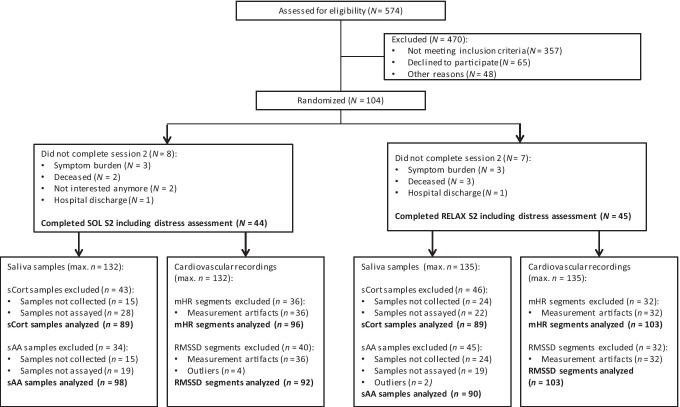
Patient and sample flow chart. SOL “Song of Life" music therapy, RELAX relaxation, S session, sCort salivary cortisol, sAA salivary α-amylase, mHR mean heart rate, RMSSD root mean square of successive differences

Between December 2018 and August 2020, a total of 574 patients were assessed for eligibility. Of the *N* = 104 patients randomized, *N* = 89 completed session two including the pre-to-post session assessment of momentary distress. Among maximally available *n* = 267 samples, *n* = 178 samples (66.6%) were finally analyzed for sCort, *n* = 188 (70.4%) for sAA, *n* = 199 (74.5%) for mHR, and *n* = 195 (73.0%) for RMSSD. Missingness in the sCort/sAA data occurred as samples could either not be collected due to xerostomia or nausea or as samples did not contain enough liquid for the assays (i.e., < 50 μl for sCort and < 10 μl for sAA). The main reason for loss of data in the PPG recordings was measurement artifacts caused by movement or reduced peripheral blood flow (Fig. 2). The patient sample for analysis consisted of mainly women (*n* = 66, 74.2%) with a mean age of *M* = 65.8 years. *n* = 87 participants (97.8%) had a primary diagnosis of advanced cancer (Table 1). There were no significant group differences for all outcome measures at T0, with the exception of mean heart rate indicating a lower mean heart rate at T0 in the SOL group (*t*(197) = 1.99, *p* = 0.05).

**Table 1:** Baseline characteristics of sample for analysis (*N* = 89)

Characteristic	SOL	RELAX
Participants per site (*n*, %)		
Study site 1	21 (23.60%)	21 (23.60%)
Study site 2	23 (25.84%)	24 (26.97%)
Age (*M*, *SD*, years)	68.07 (11.52)	63.58 (11.98)
Sex (*n*, % female)	34 (77.3%)	32 (71.1%)
Cancer type (%, *n*)		
Gastrointestinal	31.8% (14)	26.7% (12)
Gynecologic	27.3% (12)	28.9% (13)
Skin	9.1% (4)	8.9% (4)
Lymphatic	6.8% (3)	11.1% (5)
Thoracic	11.4% (5)	11.1% (5)
Other	11.4% (5)	11.1% (5)
Non-cancer	2.3% (1)	2.2% (1)
Karnofsky performance status scale (*M, SD*, 0–100)	43.86 (16.17)	50.22 (21.27)
Treatment expectancy (*M*, *SD*, 1–5)	3.74 (0.73)	3.76 (0.61)

*SOL* “Song of Life” music therapy, *RELAX* Relaxation intervention

**Fig. 3:**
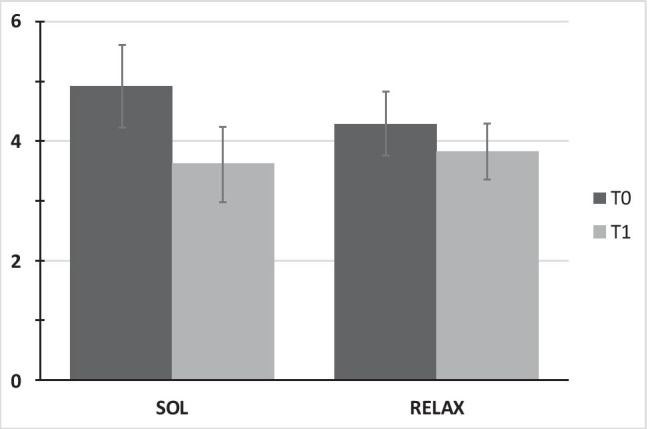
Means and standard errors of momentary distress (NCCN Distress Thermometer). SOL “Song of Life” music therapy, RELAX relaxation, T time point

Table 2 depicts the results of multilevel modeling of treatment effects. Analysis of momentary distress showed a statistically significant time*treatment interaction indicating a greater reduction for SOL participants (M1: *b* =  − 0.83, *p* = 0.02). Means and standard errors are illustrated in Fig. 3.

**Table 2:** Results of multilevel modeling with all available data (AAD) and multiply imputed data (MID)

Outcome	Distress^a^				sCort (log)^b^				sAA (log)^c^				Mean heart rate^d^							RMSSD (log)^d^			
	M1 (AAD)		M2 (MID)		M3 (AAD)		M4 (MID)		M5 (AAD)		M6 (MID)		M7 (AAD)			M8 (MID)				M9 (AAD)		M10 (MID)	
Fixed effects	Est	*p*	Est	*p*	Est	*p*	Est	*p*	Est	*p*	Est	*p*	Est		*p*		Est		*p*	Est	*p*	Est	*p*
Intercept	**4.87**	**.00**	**4.77**	**.00**	**2.29**	**.00**	**2.24**	**.00**	**3.88**	**.00**	**4.94**	**.00**	**88.17**		**.00**		**88.11**		**.00**	**2.47**	**.00**	**2.45**	**.00**
Treat	**1.44**	**.04**	1.27	.10	0.23	.33	0.28	.14	0.27	.38	0.13	.67	** − 7.89**		**.05**		− 5.54		.12	0.22	.17	0.22	.19
Time1	− **0.88**	**.00**	− **0.87**	**.00**	− **0.08**	**.00**	− **0.09**	**.00**	0.01	.81	− 0.05	.22	− **3.65**		**.03**		− 2.30		.11	0.02	.40	0.02	.42
Time2	-		-		-		-		-		-		**0.78**		**.05**		0.44		.22	-	-	-	-
Time1*Treat	− **0.83**	**.02**	− **0.75**	**.04**	− 0.04	.20	− 0.02	.50	− 0.11	.23	− 0.03	.72	6.00		.06		4.06		.18	− 0.04	.37	− 0.02	.68
Time2*Treat	-		-		-		-		-		-		− 1.32		.11		− 0.91		.23	-	-	-	-
Random effects (variances)																							
Intercept L2	4.33		4.28		0.67		0.66		0.55		1.26		142.84				131.47			0.29		0.30	
Residual variance	1.28		1.32		0.03		0.03		0.14		0.18		6.95				7.25			0.06		0.07	
Model fit																							
AIC	745.71				169.51				235.17				1267.61							212.59			
BIC	767.98				207.28				260.26				1303.84							242.05			
***N***																							
Observations (L1)	178				172				120				199							195			
Patients (L2)	89				62				47				74							72			

Bold effects were statistically significant on the level of *p* < .05

*sCort* salivary cortisol, *sAA* salivary alpha-amylase, *RMSSD* root mean square of successive differences, *M* Model, *AAD* all available data, *MID* multiply imputated data, *Est.* Estimate, *Treat* treatment (0 = relaxation intervention; 1 = “Song of Life music” therapy), *Time1* linear trend of time (0, 1, 2), *Time2* quadratic trend of time (0,1,4), *L* level, *AIC* Akaike Information Criterion, *BIC* Bayesian information criterion

^a^Distress models were statistically controlled for ‘study site’

^b^sCort models were statistically controlled for ‘study site’, ‘sedative medication’, ‘corticosteroid medication’, ‘time since last meal’, ‘sex’,‘age’

^c^sAA models were statistically controlled for ‘study site’, ‘sex’,‘age’

^d^Mean heart rate and RMSSD models were statistically controlled for ‘study site’,‘sex’, ‘age’

**Fig. 4:**
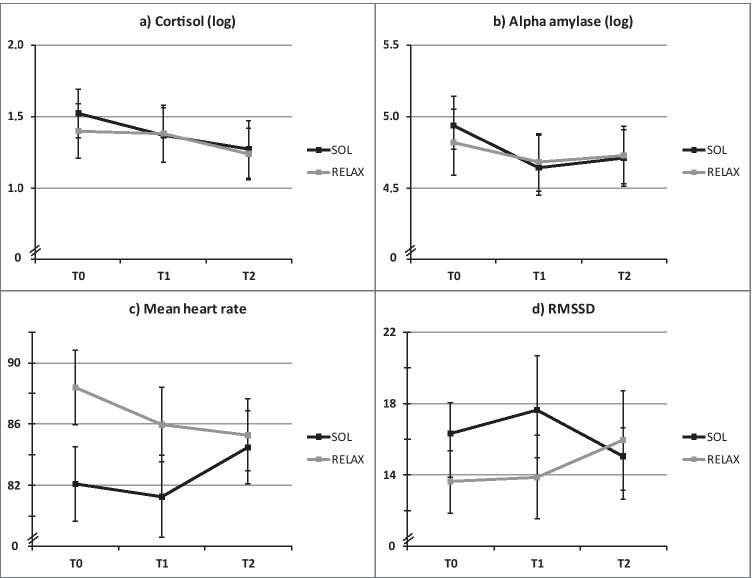
Means and standard errors of sCort, sAA, mHR, and RMSSD. sCort salivary cortisol, sAA salivary α-amylase, mHR mean heart rate, RMSSD root mean square of successive differences, SOL “Song of Life” music therapy, RELAX relaxation, T time point

Figure 4 displays means and standard errors for psychobiological data. With regard to all psychobiological outcomes, we found no statistically significant time*treatment interaction in the AAD set (all *p* > 0.05). However, multilevel modeling showed a significant main effect of time (linear) in sCort (M3: *b* =  − 0.08, *p* < 0.001) indicating that cortisol concentration decreased over time in both interventions with no differences between groups. Moreover, both the linear (M7: *b* =  − 3.65, *p* = 0.03) and quadratic trend of time (M7: *b* = 0.78, *p* = 0.05) were statistically significant for mHR, suggesting a U-shaped trajectory for both SOL and RELAX. In addition, mHR was generally higher in the RELAX than in the SOL group, which was represented by a main effect of treatment (M7: *b* =  − 7.89, *p* = 0.05).

Sensitivity analyses with MID yielded the same patterns of findings regarding the time*treatment interactions, i.e., a significant interaction effect for distress (M2: *b* =  − 0.75, *p* = 0.04) and the absence of time*treatment interactions in all psychobiological outcomes (all *p* > 0.05). MID provided further support for the observed overall linear decrease in sCort (M4: *b* =  − 0.09, *p* < 0.001). However, no significant main effect of treatment and no quadratic main effect of time were found in mHR (M8: both *p* > 0.05).

## Discussion

As one of the first studies to integrate psychobiological stress marker assessments, the present trial aimed to investigate potential working mechanisms of psychosocial interventions in palliative care by exploring differential effects of music therapy and mindfulness with regard to distress, sCort, sAA, mHR, and HRV. In line with previous research on music therapy in palliative care [30], our findings provide evidence for a reduction of self-rated momentary distress in both groups and a significantly stronger reduction in SOL participants (without significant group differences in distress at T0). One reason for the superiority of SOL might be the higher emotional involvement of SOL participants as they received a live performance of a biographically meaningful song while patients in the mindfulness group participated in a standardized mindful breathing exercise.

With regard to psychobiological outcomes, we found no differential treatment effects contrary to the hypothesis. Corresponding to previous inconsistent evidence on the psychobiological effects of psychosocial interventions in palliative care [6, 27, 31, 32], we were unable to show superiority in terms of one treatment being more efficient than the other in affecting stress marker trajectories. One of the main reasons may be the overlapping working mechanisms between the two psychosocial treatments (e.g., therapeutic alliance, attention, empathy) and the lack of a third usual-care study arm. While primary and secondary endpoints of the parent trial were tailored to the SOL intervention (i.e., questionnaires on psycho-spiritual integration), psychobiological stress markers may respond more broadly to interpersonal and empathic care, regardless of the specific technique. Accordingly, sCort and mHR decreased from T0 to T1 in both groups in terms of descriptive statistics and significant main effects of time possibly indicating an effect of both interventions although a third usual-care-only study arm would be required to test this conclusion. Another explanation for the lack of differential psychobiological effects might be associated with challenges in data collection in palliative care [24]. Although we chose the cotton swab particularly as a non-invasive assessment, a considerable number of patients was not able to use it at all or to tolerate it long enough due to xerostomia or nausea. The discrepancy between the number of samples collected and samples successfully assayed might mirror the weakened capacity of the participants’ salivary glands to produce enough liquid for analysis. Correspondingly, photoplethysmography also faced data losses due to technical problems, movement artifacts, and reduced blood flow in the patients’ limbs.

The major strength and novelty of the present study were the integration of self-ratings with markers of HPA axis and ANS reactivity in the evaluation of psychosocial interventions in palliative care. However, one particular limitation was the abovementioned high attrition rate in both salivary and photoplethysmographic sampling. We therefore analyzed data with an intention-to-treat approach using both AAD and MID in sensitivity analysis. Still, the study might have been statistically underpowered to detect small differential effects due to missing data. Of note, the different self-report and biological stress markers assessed here have individual time frames to respond (immediate response in subjective markers, mHR and sAA, more delayed responses in sCort). Therefore, an additional and later assessment might have captured potential differences between the interventions. Future research might include large-scale samples or a larger number of repeated measurements to counteract these difficulties. Another limitation was the lack of a usual-care-only group, which would have allowed for examining whether the two psychosocial interventions had more beneficial psychobiological effects than no treatment.

## Conclusion

Findings from this RCT suggest a beneficial effect of the SOL music therapy intervention on distress compared to mindfulness exercises. However, no differential treatment effects were found with regard to cortisol, α-amylase, mean heart rate, and HRV. Future studies should continue to investigate optimal psychobiological measurement methods in this field in order to complement the evaluation of effectiveness of psychosocial treatments on a subjective level.

## Data Availability

The dataset for this study will be made available on request from the corresponding author.

## Code availability

The code will be made available on request from the corresponding author.
